# *Mycobacterium tuberculosis* Infection following Kidney Transplantation

**DOI:** 10.1155/2013/347103

**Published:** 2013-10-08

**Authors:** Karima Boubaker, Tahar Gargah, Ezzedine Abderrahim, Taieb Ben Abdallah, Adel Kheder

**Affiliations:** ^1^Internal Medecine Department, Charles Nicole Hospital, Boulevard 9 Avril, Bab Souika, 1006 Tunis, Tunisia; ^2^Pediatric Department, Charles Nicole Hospital, Tunisia; ^3^Research Laboratory of Immunology (LR03SP01), Tunisia

## Abstract

*Introduction and Aims*. Post-transplant tuberculosis (TB) is a problem in successful long-term outcome of renal transplantation recipients. Our objective was to describe the pattern and risk factors of TB infection and the prognosis in our transplant recipients. *Patients and Methods*. This study was a retrospective review of the records of 491 renal transplant recipients in our hospital during the period from January 1986 to December 2009. The demographic data, transplant characteristics, clinical manifestations, diagnostic criteria, treatment protocol, and long-term outcome of this cohort of patients were analyzed. *Results*. 16 patients (3,2%) developed post-transplant TB with a mean age of 32,5 ± 12,7 (range: 13–60) years and a mean post-transplant period of 36,6months (range: 12,3 months–15,9 years). The forms of the diseases were pulmonary in 10/16 (62,6%), disseminated in 3/16 (18,7%), and extrapulmonary in 3/16 (18,7%). Graft dysfunction was observed in 7 cases (43,7%) with tissue-proof acute rejection in 3 cases and loss of the graft in 4 cases. Hepatotoxicity developed in 3 patients (18,7%) during treatment. Recurrences were observed in 4 cases after early stop of treatment. Two patients (12.5%) died. *Conclusion*. Extra pulmonary and disseminated tuberculosis were observed in third of our patients. More than 9months of treatment may be necessary to prevent recurrence.

## 1. Introduction and Aims 

Tuberculosis (TB) is an opportunist infectious disease with obligatory declaration, caused by *Mycobacterium tuberculosis* discovered by German Robert Koch in 1882 from where the name bacillus of Koch (abbreviation BK) is derived.

TB is the most important infectious disease in humans and is endemic in many developing countries [1, 2], with a prevalence estimated at 27,07/100 000 inhabitants in 1995 in Tunisia [3]. In situations wherein the immune system becomes impaired such as acquired human deficiency syndrome (AIDS), chronic renal failure, or organ transplant recipients treated by immunosuppressive drugs, TB is a major problem and the key to controlling is rapid detection.

The TB incidence in kidney recipient patients is 20 to 74 times greater than that among the general population [4]. This is due to iatrogenic immunosuppression in transplant recipients which accounts for a progressive impairment in cellular immune function allowing the development of BK which is an intracellular germ [5, 6]. Posttransplant TB is a problem in successful long-term outcome of kidney transplant recipients and is a life-threatening infection. However, its diagnosis is often delayed.

With the emergence of newer potent immunosuppressive regimens and an increased incidence of TB in the general population, TB among kidney transplant recipients can be anticipated. 

This study tried to examine the prevalence, course, and outcome of TB in our kidney transplant recipients.

## 2. Patients and Methods

### 2.1. Patients 

In this retrospective study, we reviewed medical records of 491 renal transplant recipients in our department from June 1986, date of the first kidney transplantation, to December 2009. 

The criteria of exclusion were onset of tuberculosis before kidney transplantation or after 3 months of the return in dialysis. 

Sixteen patients received treatment for TB. Diagnosis of TB was made on bacteriological, histological, and/or therapeutic proof or in front of the association of clinical, biological, and/or radiological elements of presumption. 

### 2.2. Methods

The bacteriological analysis includeed using direct light microscopy to reveal acid-fastbacilli (AFB) in at least 1 Ziehl-Neelsen-stained respiratory tract secretion, urine or other biological liquid sample or positive cultures for the etiologic pathogen on a special medium of Lowenstein or one of its multiple alternatives (Jensen, Coletsos, etc.).

The histological analysis was the presence of a gigantic-cellular granuloma with necrosis caseous on the liquid of puncture or a fragment coming from an organ biopsy. 

The following data were obtained from each patient's medical record: patient demographics (age and sex), presence of another comorbid disease or preexisting risk-factors for TB infection, symptoms (fever, cough, impairment of general state), urine exam, biology (creatinemia, biological inflammatory syndrome, and complete blood count), chest radiograph patterns, organ involvement, diagnostic methods, administration of anti-TB therapy, and mortality. 

Radiographic patterns were classified as normal findings, miliary pattern, pleural effusion, parenchymal cavitation, nodules, pulmonary infiltrate, and hilar or mediastinal lymphadenopathy. As the association of radiographic patterns is possible, this makes the sum of the frequencies of radiographic patterns be more than 100% [7, 8].

A search for confections with *Candida albicans*, *Pseudomonas aeruginosa*, *Staphylococcus aureus*, *Acinetobacter haemolyticus*, *Cytomegalovirus*, and/or *Aspergillus* was done. 

Interval between diagnosis of TB and date of kidney transplantation and circumstances of discovery of TB for each patient were recorded. 

Mendel-Mantoux skin testing was carried out by the intracutaneous inoculation of purified protein obtained from vaccine BCG and called tuberculin into the volar surface of the forearm [7]. The test is read after 72 hours and is positive if induration is ≥10 millimeters. 

A disseminated TB was defined when 2 organs were involved.

Results were analyzed using Statview 5.0 software. Values were expressed as mean ± standard deviation. 

Our 16 patients were compared with 29 controls who were matched for age, sex, and type of dialysis and who were transplanted at the same period. 

The groups were compared as for time spite on dialysis, allograft dysfunction and number of acute rejection.

## 3. Results

The characteristics of the 2 groups (TB group and control group) were summarized in Table 1. 

Sixteen patients (3,2%) developed posttransplant TB. The overall incidence of TB was 72/100 kidney transplant recipient/year (Table 2).

They were 14 men and 2 women. Mean age was 32,5 ± 12,7 (range: 13–60) years. Median age was 34 years and 62% of patients were aged more than 30 years. 

A previous history of urogenital TB was found in 1 case and direct contact with a TB carrier in 2 cases. Blood group was A in 2 cases, B in 1 case, AB in 3 cases, and O in 10 cases. 

Causes of end stage renal stage were glomerulonephritis in 5 cases, diabetic nephropathy in 1 case, lupus nephritis in 1 case, interstitial nephritis in 4 cases, hypertension in 1 case, and unknown in 4 cases. Time spent on dialysis was 38,6 months (10,3 months–21,1 years). It is significantly higher than controls (38,6 years versus 27,4 years, *P* = 0,27). Initial immunosuppressive regimen associated antilymphocyte serum in 10 cases and steroids in all cases. Maintenance immunosuppressive regimen associated before diagnosis of TB, steroids in all cases, cyclosporine in 4 cases, tacrolimus in 2 cases, mycophenolate mofetil in 7 cases, and azathioprine in 7 cases. 

Diabetes was observed in 3 cases and hepatitis C in 4 cases. Seven patients presented an acute rejection before diagnosis of TB. There was only one episode of acute rejection in 5 cases and 2 episodes in 1 case.

TB patients were not significantly different from controls by means of diabetes and acute rejection.

Mean interval between kidney transplantation and TB diagnosis was 36,6 months (range: 12,3 months–15,9 years) with median of 23,6 months.

Clinical picture associated unexplained and moderate fever in 15 cases (93,7%), pleuritic syndrome in 3 cases, and a pulmonary infection resistant to antibiotics in 1 case. 

At biology, sterile leukocyturia was noted in 2 cases, graft dysfunction in 5 cases, biological inflammatory syndrome in 12 cases, and pancytopenia in 1 case. 

Bacteriological analysis confirmed TB diagnosis in 9 cases (AFB at direct light microscopy in 7 cases, positive culture in 9 cases).

A confection with *Candida albicans* was found in 1 case, with *Cytomegalovirus* in 1 case and with *Aspergillus* in another case. 

Tuberculin skin test done in 5 cases was positive in 2 cases. 

**Figure 1:**
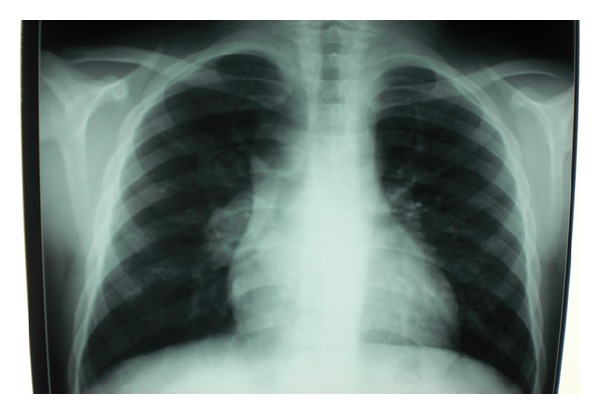
Chest X ray showing mediastinal enlargement.

**Figure 2:**
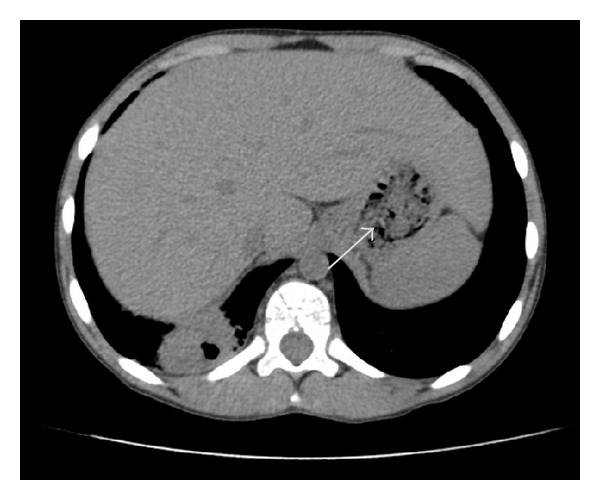
Chest computed tomography showing cavitation's of the left lung.

Radiographic patterns showed abnormalities in all cases with miliary pattern in 3 cases, pleural effusion in 5 cases, cavitation in 1 case, nodules in 2 cases, pulmonary infiltrate in 6 cases, mediastinal lymphadenopathy in 2 cases, and spondylodiscitis L5 in 1 case (Figures 1 and 2). 

Diagnosis of tuberculosis was confirmed only in 14 cases, on bacteriological proof in 9 cases and on histological proof in 5 cases. 

Pulmonary localization of TB was the most frequent observed in 62,6% of cases. Extra pulmonary localization was observed in 3 cases (18,7%) and disseminated TB in 3 cases (18,7%) (Table 3).

All patients initially received 4-drug combination therapy which associated isoniazid, rifampicin, ethambutol, and pyrazinamide during 2 months relayed and then a daily therapy by isoniazid and rifampicin. The average total duration of the treatment was 10,3 ± 3,5 months (1–17 months) (Table 4). 

Because of drug interaction, an increase in the dose of calcineurin inhibitor and steroid was done in 2 cases and in steroids alone in 1 case. 

All patients were followed up. After a mean followup of 291,3 months (88–755 months), recovery of TB was obtained in 8 cases and graft dysfunction in 7 cases (43,7%) with tissue-proof acute rejection in 3 cases and loss of the graft in 4 cases (Table 4).

Hepatotoxicity observed in 3 cases and hyperuricemia in 4 cases were reversible after stop of treatment. 

Death was observed in 2 patients (12.5%) and was related to tuberculosis meningitis in one case and to severe sepsis in the other case (Table 4).

TB patients were not significantly different from controls by means of graft and patient survival.

Recurrence of TB was observed in 4 cases after early stop of treatment.

The first patient in whom there is no proof of tuberculosis and who was treated with anti-TB therapy had recurrence at the same localization (vertebra) needing another TB therapy for 12 months. After a followup of 9,3 months, the patient had lost its graft and was in hemodialysis.

The second patient in whom there is no proof of pleural tuberculosis and who was treated with anti-TB therapy is recovered after 12 months of anti-TB therapy with normalization of the chest X-ray.

## 4. Discussion

TB in the kidney transplant recipients in our department displayed the following characteristics. 

High incidence within a short time after transplantation with 50% of patients was diagnosed within the first 2 years after-transplant, high coinfection rate (18,7%). Fever was the most common clinical manifestation (93,7%). Graft dysfunction (43,7%), liver function damage (18,7%) and hyperuricemia (25%) were the main adverse effects of anti-TB treatment. Mortality of patients reached up to 12,5 %.

We found that prevalence of TB was 3,2%, lower to the prevalence observed in developing countries (11,8 to 13,3%) [4, 8]. Prevalence of latent tuberculosis is even higher [9]. 

TB incidence was 72/100 kidney transplant recipient/year, 25-fold higher than among the Tunisian population (17/100 000 inhabitant/year) [10]. It reaches the incidence observed in developing countries which is 20- to 74-fold higher that than among the general population [4, 8]. 

Annual incidence of TB is 0.47% among kidney transplant recipients [4]. 

Posttransplantation TB is predominantly the result of reactivation of an earlier quiescent TB focus [11] with an exudative form during the early posttransplantation period [2]. Then, chronic renal failure patients who are awaiting transplantation should be carefully evaluated for previous TB anamnesis and family history. Rarely, in less than five percent of patients, TB is caused by nosocomial acquisition or donor transmission [12, 13]. 

Mean age of our patients was 32,5 years, versus data of the literature which is 37,7 years [14]. No difference in age or gender between kidney transplant recipients with or without TB is described [14]. 

Time spent on dialysis was 38,6 months versus data of the literature which is 30,3 months and it is significantly higher compared to kidney transplant recipients without TB [14]. 

Half of our patients developed TB before the end of their second year of transplantation. In fact, the peak incidence is after the first year of transplantation [15, 16].

Risk factors of TB transmission to kidney transplant recipients are direct contact with a TB carrier [17], blood group AB [18], hepatitis C [19], and allograft dysfunction with creatininemia higher than 1.5 mg/dL [14, 19]. 

Prolonged duration of pretransplant hemodialysis is associated with increased risk of developing TB because and of uremia altered phagocytosis, bactericidal activity, and lymphocyte transformation. However, it was not been found as a risk factor in our study.

Previous history of TB is controversial in the development of post kidney transplantation TB [14, 17]. However, in some studies, 9,5% to 13.5% of kidney transplant recipients had previous history of TB [4, 20].

Diabetes and more than 3 episodes of acute rejection were not found as risk factors of TB in our study.

Immunosuppressive drugs used in these patients explain the increased incidence of TB [14]. Higher doses of steroids prescribed for long course [21], mycophenolate mofetil more than one year [2] in switch to azathioprine [22], tacrolimus [18, 23], and antilymphocyte serum [21] are associated with high risk of TB. However, Campath (alemtuzumab) does not increase the incidence of TB [24].

The clinical features of TB can be unusual and may be masked by the blunted response to infection. Common clinical abnormalities include pyrexia, pulmonary infiltrates, exudative pleural effusion, and exudative ascites. In our study, moderate and permanent fever of unknown origin was observed in 93,7% of cases versus 71% to 82,9% in the literature [4, 25–27]. Impairment of the general state was observed in 31,2% patients in our study versus 40% in literature [27, 28]. 

Pulmonary TB was observed in 62,6% of our patients. It continues to be the most common form in kidney transplant recipients [29]. Pulmonary signs were observed in 37,5% of the cases particularly coughing (12,5% of the patients) versus 56.1% in the literature accompanied by spittle in 39% of the cases [26]. No case of hemoptysis was reported in our study while they are observed in 20% in other studies [30].

Chest X-ray is abnormal in 81,2% of our patients showing pulmonary infiltrates in 37,5% of cases versus 60% in the literature, nodules, cavities in 6,2% of cases versus 10% in the literature, miliary pattern, pleural effusion, mediastinal lymphadenopathy, and/or spondylodiscitis [4, 31]. 

Extra pulmonary presentations of TB are more frequent in kidney transplant recipients compared to immunocompetent patients, observed in 18,7% of cases in our study versus 28.6 to 50% in other studies [4, 32, 33]. Extra pulmonary symptoms are sometimes atypical such as an unusual gastrointestinal symptomatology, skin lesions not improved by antibiotics, and/or dissemination [2, 16, 31]. 

Genitourinary TB that occurs after kidney transplantation is uncommon and appears to present differently than genitourinary TB in the nontransplant population [31, 35, 36]. It has a different clinic radiological presentation with predominance of systemic symptoms, disseminated TB, multiple parenchymatous renal foci, and lower frequency of lesions of the collecting system [31]. 

Predominantly parenchymatous renal involvement was more frequent in immunocompromised patients, who also had lower frequency of stenosis of the collecting system and contracted bladder [31, 37]. 

Genitourinary symptoms are more likely to be found in immunocompetent patients with TB of the renal system than in immunocompromised hosts. Our 2 kidney transplant recipients with genitourinary TB did not present with urinary symptoms. They had only fever and sterile leukocyturia. 

TB localized to the renal allograft is an unusual presentation of TB and may be the cause of graft rejection and loss [38]. The allograft biopsy is helpful when other investigations are inconclusive with symptoms of allograft dysfunction [2]. Histology shows, in this form, granuloma suggestive of TB [2, 25, 39]. 

Cerebral TB can be revealed by an intracranial hemorrhage [40]. In our case of meningeal TB, the patient presented confusion. 

Disseminated TB is 3 times more frequent in kidney transplant recipients compared to patients without immunosuppression, accounting for 18,7% of cases in our study and 23.8 to 62.5% of cases in other studies [4, 5, 31, 38]. This increased frequency of disseminated TB is explained by the fact that, in the context of immunosuppression, TB behaves as a severe bacterial infection, with bacteremia and visceral metastatic foci [31]. 

75% of our patients had biological inflammatory syndrome. The measurement of C reactive protein which is a protein of the inflammation levels may be a useful tool for differentiating bacterial or TB infection from CMV infection in kidney transplant recipients. Patients with TB and bacterial infection presented lower levels of CRP than patients with CMV disease [41].

In our study, a bacteriological or histological confirmation was obtained in 75% of the cases. A treatment with quinolones, which is a second line anti-TB drugs, can negative AFB at Ziehl-Neelsen-stained smear using direct light microscopy [2]. 

Indeed, only a positive culture of BK confirms the diagnosis of TB in 35,71% of the cases [42] because we cannot differentiate between acid-fast bacilli (AFB) and atypical mycobacterium at Ziehl-Neelsen-stained smear. However, only one AFB in only one field is enough with the startup to the antiTB treatment while waiting for the culture. 

Tuberculin skin test is not helpful in the majority of patients because it has low sensitivity and specificity. Low sensitivity of 50% for predicting posttransplant TB is explained by anergy due to deterioration of cellular immunity particularly in poor-nourished and anemic patients, males, elderly, smokers, patients with hepatic pathology, peptic ulcer, and/or prolonged duration of pretransplant hemodialysis [43–46]. Sensitivity of skin test increases to 75% in kidney transplant recipients after exclusion of patients with anergy [2, 9, 26]. The sensitivity of the skin test is not affected by bacillus-Calmette-Guerin (BCG) vaccine [43]. Low specificity of 52% for predicting posttransplant TB is explained by higher positivity of the test in the endemic countries [9, 26, 43]. 

Given that we are an endemic country of TB, and to increase sensitivity and specificity, it is necessary to increase doses of tuberculin at 10 units [7] and repeat the skin test if the first injection or the reading is not satisfactory [47]. Nutritional status (hemoglobin, albumin, and creatinine) should be improved and time spent on dialysis should be reduced [43]. Moreover, to increase the skin test specificity by distinguishing between latent TB infection from BCG-induced T-cell reactivity towards early secretory antigenic target-6 (ESAT-6), a protein specific for *Mycobacterium tuberculosis *but absent from the BCG-vaccine strain is found in 52.9% of all individuals with purified protein-derivative (PPD) reactivity in vitro [9].

The diagnosis of genitourinary TB is made by urine cultures done for the detection of mycobacteria. Because of the delay inherent in diagnosis by culture, rapid testing methods for identification of *Mycobacterium tuberculosis*, such as polymerase chain reaction analysis of the urines which made diagnosis of TB in 17.86% of the cases or DNA probing of urine, should be employed [29, 42].

Aggressive investigations must be done in patients with pyrexia, pulmonary abnormalities, scanty sputum, and weight loss and whose diagnosis was not confirmed by bacteriology [11, 48]. X-ray and computed tomography scan with puncture and/or biopsy of the chest should be done in such cases (Figures 1 and 2). 

A confection with *Candida albicans*, *Cytomegalovirus*, and *Aspergillus* was observed in 18,7% of cases. It was observed in 19,5% of cases inliterature. Other confections with *Pseudomonas aeruginosa*, *Staphylococcus aureus*, and *Acinetobacter haemolyticus* are also observed [26, 49, 50].

The treatment of TB in kidney transplant recipients should be the same as in the general population [11, 42, 51, 52]. rifampicin is an important TB treatment and was prescribed in all our patients. However, its use must be undertaken with caution because of its frequent interaction with immunosuppressive drugs, and blood levels of immunosuppressive drugs should be monitored. 

Prolonged followup should be provided. Patients can show good clinical and radiological responses under therapy but complications are possible related either TB or side effects of antibacterial drugs [21]. 

Six patients (37,5%) were successfully treated with quadruple anti-TB therapy for 12 months (9–17 months). Anti-TB treatment can induce a successful management with reduction of allograft nephropathy, graft nephrectomy, and mortality [2, 25, 53, 54]. Response to antiTB treatment should be considered to make a diagnosis among patients highly suspected of TB infections.

However, several complications of antiTB treatment can appear. 

Acute rejection is observed in 18,7% in our study and in 29.3% of cases in the literature [11]. It can be seen even after the stop of the anti-TB treatment [21]. To avoid acute rejection, blood levels of calcineurin inhibitors should be monitored closely with an increase in doses in 53.57% to 100% and antilymphocyte globulin can be used as antirejection prophylaxis [11, 21, 28, 30, 42].

Chronic allograft nephropathy is a serious complication observed in 65% of the cases and has a negative impact on the graft survival [2, 20, 39, 55]. 

Loss of the graft was observed in 4 cases in our study (25% of cases).

Hepatoxicity is a considerable risk of treatment observed in 3 cases (18,7%) in our study and in 17.1% to 42.8% of the cases in the literature, as a result of additive toxic effects of immunosuppressive drugs particularly isoniazid [20, 28, 42]. Hepatitis needs close observation because of the frequent occurrence of viral hepatitis in such cases.

Hyperuricemia reversible after stop of treatment was found in 4 cases (25%) in our study.

Recurrence of TB is a frequent complication among kidney transplant recipients [33]. More than 9 months of treatment may be necessary to prevent recurrence [21, 42, 53, 56–58]. 

Two patients (12,5%) died due to TB-related complications in our study and 12.9% to more than 22% of cases in other studies [21, 26, 55]. Mortality is higher when TB occurs during the first year after kidney transplantation, among poor-nourished patients, treated with steroids and having hypoxia [59]. 

Death was observed in 2 patients (12.5%) and was related to tuberculosis meningitis in one case and to severe sepsis in the other case. The first patient had presented meningeal and vertebral TB after stop of treatment. The second patient had chronic allograft dysfunction with severe renal insufficiency.

Prophylaxis is recommended for high-risk patients with previous history of TB before kidney transplantation and direct contact with a TB carrier. It associated isoniazid at a daily dose of 300 mg for patients weighing more than 35 kg and 5 mg/kg in patients weighing less than 35 kg, and pyridoxine at the dose of 50 mg daily for 1 year [11, 17, 48, 55].

## 5. Conclusion

Tunisian kidney transplant recipients face a high risk of TB because of their immunecompromised state and epidemiological prevalence of the disease. Its clinical presentation is atypical with a high frequency of the extra pulmonary and disseminated localizations observed in third of cases in our patients. Therefore, attention should be given to this differential diagnosis in clinical practice. 

To prevent recurrence of TB, which was frequent (18,7% of cases), prolonged antiTB treatment for at least 9 months is recommended.

## Figures and Tables

**Table 1:** Characteristics of TB and control groups.

	TB group	Control group	*P*
Donor age (years)	32,5	28,7	0,2843
Recipients sex ratio (M/F)	14/2	26/3	>0,9999
Type of dialysis	PD = 2 HD = 12 HD_PD = 2	PD = 4 HD = 24 HD_PD = 1	0,5072

HD: hemodialysis, F: female, M: male, PD: peritoneal dialysis, TB: tuberculosis.

**Table 2:** Epidemiological, clinical, and biological characteristics of TB kidney recipient's patients before diagnosis of TB.

Name	Sex	Age	Previous history of TB and direct contact with a TB carrier	Nephropathy	Time spent on dialysis (years)	Donor	Immunosuppressive regimen	AR	Ttt of AR	HC	Diabetes	Creat *μ*mo/L
(1) A M	F	14	—	Unknown	39,688	Cadaver 38 years	CS + MMF	1	ALS + CS	Non	Non	178
(2) A H	M	32	—	Interstitial	25,068	Mother 61 years	CS + AZT	0	—	Non	Non	140
(3) Z A	M	42	—	Glomerular	39,951	Brother 50 years	CS + AZT	1	ALS + CS	Non	Non	164
(4) Gh N	F	34	Husband	Interstitial	23,359	Mother 65 years	CS + AZT	1	ALS + CS	Non	Non	157
(5) D Y	M	60	—	Diabetic	25,823	Wife 54 years	CS + MMF	0	—	No	Yes	150
(6) H O	M	22	—	Lupic	14,324	Sister 39 years	CS + MMF	0	—	No	No	128
(7) D M	M	34	—	Glomerular	13,996	Sister 32 years	CS + ciclo + AZT	0	—	No	No	90
(8) H A	M	22	—	Interstitial	31,836	Mother 57 years	CS + tacrolimus + MMF	0	—	No	No	128
(9) M A	M	51	Urogenital	Unknown	99,745	Brother 30 years	CS + ciclo	0	—	Yes	No	96
(10) M F	M	27	—	Hypertension	17,018	Mother 46 years	CS + AZT	2	ALS + CS CS	No	No	520
(11) H Dh	M	13	—	Interstitial	25,462	Cadaver 27 years	CS + ciclo + MMF	0	—	No	No	118
(12) M A	M	19	Brother	Unknown	20,337	Mother 40 years	CS + MMF	0	—	No	No	90
(13) J K	M	37	—	Unknown	188,386	Sister 43 years	CS + tacrolimus + MMF	1	ALS + CS EP	Yes	No	142
(14) Ch N	M	39	—	Glomerular	18,957	Brother 34 years	CS + AZT	1	ALS + CS	No	No	113
(15) B F	M	36	—	Glomerular	18,858	Sister 30 years	CS + ciclo + AZT	1	ALS + CS	Yes	Yes	111
(16) J H	M	38	—	Glomerular	22,045	Sister 36 years	CS + MMF	0	—	Yes	Yes	187

ALS: antilymphocyte serum, AR: acute rejection, AZT: azathioprine, ciclo: cyclosporine, Creat: creatininemia, CS: steroids, F: female, HC: hepatitis C infection, M: male, MMF: mycophenolate mofetil, TB: tuberculosis, ttt: treatment.

**Table 3:** Interval between tuberculosis diagnosis and kidney transplantation, clinical and para clinical picture, proof and localization.

Name	Interval KT/TB (years)	Circumstances of discovery and clinical picture	Biology	Creat *μ*mo/l	Radiology	Proof	Localization(s)
(1) A M	9,561	Fever sweat low back pain	BIS	227	Spondylodiscitis L5	0	Vertebra
(2) A H	13,339	Fever impairment of general state	ARF	170	Pulmonary infiltrate pleuritic effusion	Bacteriological	Urinary and pulmonary
(3) Z A	253,503	Fever	ARF	500	Pulmonary infiltrate	Bacteriological	Pulmonary
(4) Gh N	62,489	Fever, impairment of general state pleuritic syndrome	BIS	134	Miliary pleuritic effusion	Histological	Pulmonary
(5) D Y	28,452	—	BIS	147	Nodules	Histological	Pulmonary
(6) H O	9,396	Fever, impairment of general state pleuritic syndrome	BIS	115	Pleuritic effusion	Histological	Pleural
(7) D M	6,505	Fever	Pancytopenia	100	Normal	Histological	Lymph nodes
(8) H A	7,984	Fever chest pain pleuritic syndrome	BIS ARF	164	Pleuritic effusion	Histological	Pulmonary
(9) M A	3,154	Fever impairment of general state sweat	BIS sterile leukocyturia	98	Hilary calcification	Bacteriological	Urinary
(10) M F	164,271	Fever, sweat chest pain	SIB	472	Pulmonary infiltrate	Bacteriological	Pulmonary and meningeal
(11) H Dh	2,825	Fever	SIB anemia	101	Nodule pulmonary infiltrate mediastina lymphadenopathy	Bacteriological	Pulmonary
(12) M A	79,047	Fever, cough, sweat impairment of general state	SIB	114	Pleuritic effusion	0	Pleural
(13) J K	1,544	Fever	Sterile leukocyturia ARF, BIS	177	Normal	Bacteriological	Pulmonary and urinary
(14) Ch N	117,257	Fever	ARF	288	Mediastinal lymphadenopathy	Bacteriological	Pulmonary
(15) B F	3,811	Fever cough pulmonary infection resistant to AB	BIS	112	Nodule miliary	Bacteriological	Pulmonary
(16) J H	93,700	Fever	BIS	400	Cavern pulmonary infiltrate	Bacteriological	Pulmonary

AB: antibiotics, ARF: acute renal failure, BIS: biological inflammatory syndrome, Creat: cretininemia, KT: kidney transplantation, TB: tuberculosis.

**Table 4:** Anti-TB treatment and course of patients.

Name	Duration of ttt TB treatment (months)	Course	Recurrence of TB	Interval between stop of TB ttt and recurrence	Duration of resumption of antiTB treatment (months)	Followup (months)	Course
(1) A M	6	ARF, DCG loss of graft	Lumbar pain and radiologic abnormalities		12	9,363	HD
(2) A H	12	Hepatotoxicity hyperuricemia	Lymph nodes TB		12	213,717	Recovery
(3) Z A	12	ARF CAD	—		—	11,992	HD
(4) Gh N	12	—	Meningeal and vertebral TB after stop of ttt		—	23,918	Death
(5) D Y	10	ARF, CAD	—		—	26,809	CAD
(6) H O	12	—	—		—	1,150	Recovery
(7) D M	6	Hepatotoxicity	Lymph nodes TB, 6 months after stop of ttt		12	149,881	Recovery
(8) H A	12	ARF	—		—	20,337	Recovery
(9) M A	10	Hepatotoxicity Hyperuricemia	—		—	58,251	Recovery
(10) M F	1	CAD	—		—	1,577	Death
(11) H Dh	9	—	—		—	9,626	Recovery
(12) M A	12	Hyperuricemia	—		—	16,657	Recovery
(13) J K	12	ARF CAD	—		—	35,055	CAD
(14) Ch N	12	CAD	—		—	47,441	HD
(15) B F	17	ARF	—		—	173,602	Recovery
(16) J H	10	Hyperuricemia ARF CAD	—		—	18,201	HD

ARF: acute renal failure, AR: acute rejection, CAD: chronic allograft dysfunction, HD: hemodialysis, ttt: TB treatment, TB: tuberculosis.
